# The Quantitative Measurement of Reversible Acute Depression after Subthalamic Deep Brain Stimulation in a Patient with Parkinson Disease

**DOI:** 10.1155/2015/165846

**Published:** 2015-05-18

**Authors:** Daniel B. Simmons, Khashayar Dashtipour

**Affiliations:** Department of Neurology, School of Medicine, Loma Linda University, Loma Linda, CA 92354, USA

## Abstract

*Background*. Depression is the most commonly reported mood symptom affecting 2–8% of patients after deep brain stimulation (DBS). Usually, symptoms develop gradually; however there have been cases of reproducible events that the mood symptoms were elicited within seconds to minutes after stimulation and were immediately reversible upon cessation of the stimulus. In the current study, we applied a self-reported questionnaire to assess the patient's mood state. *Objective*. To objectively measure the reversible acute depression induced by DBS in a patient with Parkinson disease (PD). *Methods*. A statistically validated Spanish version of the Beck Depression Inventory Short Form (BDI-SF) was used. The questionnaire was administered three times. *Results*. The patient became acutely depressed within ninety seconds of monopolar stimulation on the right side. His symptoms resolved immediately after changing the setting to bipolar stimulation. The BDI-SF scores during stimulation off, on, and off again were 15, 19, and 6, respectively. *Conclusions*. The BDI-SF scores increased during stimulation and decreased after cessation. This is consistent with a reversible depressive state. The poststimulation BDI-SF score decreased to less than half of the baseline score. This may suggest that the depression was more severe than the patient was able to express during the stimulation.

## 1. Introduction

Parkinson disease (PD) is well known for its characteristic motor symptoms consisting of bradykinesia, rigidity, resting tremor, and gait impairment. More recently, the neuropsychiatric aspects of PD have increasingly been recognized as the core pathology of the disease. Multiple patients with PD commonly suffer from depression, psychosis, anxiety, apathy, and sleep disturbances [1]. Depression is among the most prominent of these symptoms, affecting an estimated 31–35% of PD patients [2, 3]. Deep brain stimulation (DBS) of the subthalamic nucleus (STN) is well established as an effective treatment for motor complications of PD, particularly dyskinesias and motor fluctuation. However, the effect of STN DBS on mood is less consistent and there is conflicting evidence in the literature regarding long term psychiatric effects from DBS and the etiology of psychiatric changes is uncertain. A recent meta-analysis showed that 10 of 18 studies (55%) reported improvement on the mentation, behavior, and mood (MBM) subscale of the Unified Parkinson's Disease Rating Scale (UPDRS), while six studies showed worsening (33%), and two showed no change [4]. By contrast, a systematic review of behavioral changes after STN DBS reported an overall decline in MBM scores by 27% [5]. One longitudinal study of 33 patients showed initial improvements in mood and psychosocial functioning but a subsequent decline to baseline at three years [6]. A different longitudinal study of 60 patients showed clear improvement on Beck Depression Inventory (BDI) scores after three years [7].

Depressive symptoms occur in 2–8% of patients after STN DBS; mania and hypomania are the next most common psychiatric side effects occurring in 1–4%. Psychosis, anxiety, apathy, and hypersexuality account for less than 1-2% each [4, 5]. In the majority of these cases, the patients' behavioral changes develop over the course of weeks to months and are the result of chronic (rather than acute) subthalamic stimulation. A study of 22 PD patients reported a significant increase in mean apathy scores three months after STN DBS surgery [8]. A separate study reported seven PD patients who developed transient depressive episodes several months after surgery. The authors pointed out that this generally corresponded to a reduction in levodopa therapy and could usually be reversed with adjustments of medication [9].

On the other hand, there have been case reports of acute, reversible psychiatric disturbances that were linked both temporally and anatomically to specific DBS parameters. Cases of severe depression with or without suicidal ideation [10–15], mania [16, 17], hypomania [18], and uncontrollable mirthful laughter [19] and one case of intraoperative aggressive behavior [20] have been described in this context. In our case report, we are primarily concerned with acute depression. This interesting phenomenon had been previously characterized with the use of PET [10] and functional MRI [13] technology, but there had not yet been, to our knowledge, an objective psychological measure used to evaluate the patient's depressive state during the stimulation.

The following case report will describe another such patient with PD who became acutely dysphoric during the initial STN DBS programming in the postoperative period. To evaluate the patient's mood state, we administered a self-report questionnaire during three separate conditions while the patient was off, on, and off subthalamic stimulation.

## 2. Case Report

A 54-year-old right-handed Spanish-speaking male with a ten-year history of PD suffered from severe bradykinesia with freezing, severe rigidity, mild lower extremity tremor, and severe dyskinesias of all extremities. His medication regimen consisted of carbidopa-levodopa with a total dose of 600 mg levodopa per day, ropinirole XL 2 mg daily, and selegiline 5 mg twice daily. He had no history of depression or other psychiatric problems. The patient underwent the implantation of quadripolar electrodes in bilateral subthalamic nuclei under local anesthesia. The contacts on the left electrode were numbered 0 to 3, with #0 on the ventral end and #3 on the dorsal end. The contacts on the right electrode were numbered 8 to 11, with #8 ventral and #11 dorsal. Correct placement was ensured using intraoperative microelectrode recording and frame-based stereotaxy. Postoperative MRI confirmed the location of the electrodes to be in the subthalamic nuclei bilaterally. Two days later, a Medtronic PC pulse generator (Model #37601) was placed in the chest wall and was connected to the electrodes via a connection wire. There were no complications in either surgery. The patient was seen in clinic for the initial programming of the left and right electrodes on postoperative days 8 and 12, respectively. Optimal stimulation conditions were determined by gradually increasing the voltage by increments of 0.5 V with a constant pulse width of 90 *μ*sec and frequency of 180 Hz and by systematically moving up the electrode from ventral to dorsal contacts.

The left and right DBS electrodes were programmed separately, so that while one electrode was modified, the contralateral electrode was turned off. The left electrode was programmed first where its final setting was (C+, 1−), 1.8 V, 90 *μ*s, and 180 Hz. On the right side (left electrode off), monopolar stimulation through contacts #8 and #9 produced no significant improvements in motor symptoms. Stimulation through contact #10 with 2.5 V, 90 *μ*sec, and 180 Hz produced dramatic reductions in rigidity and bradykinesia; however, after two to three minutes, the patient became visibly upset. His facial expression became sorrowful and he began to cry, covering his eyes with his hand. When asked why he was upset, he did not know. It seemed difficult for him to answer our questions. His sister, who was present in the room, told us that this was very unusual for him. Within seconds of turning off the stimulation, the patient's sadness resolved and his mood returned to normal. He recalled the entire episode but could not give an explanation for his change of behavior. Stimulation with the same parameters through contact #11 elicited an identical response and resolved just as quickly as the first episode. Bipolar stimulation through contacts #10 and #11 (1.8 V, 90 *μ*sec, and 180 Hz) produced good motor improvement without mood changes or side effects.

At a ten-month follow-up visit, the patient consented to a purposeful induction of a brief depressive state using deep brain stimulation. It should be noted that approximately one month after surgery, he developed symptoms of depression for which he was prescribed an antidepressant agent. His medications at this time included carbidopa-levodopa 25/100 mg, two tablets four times per day, carbidopa-levodopa CR 50/200 mg at night, rasagiline 1 mg daily, and citalopram 20 mg daily. His current DBS settings on the left were (C+, 1−), 3.5 V, 90 *μ*sec, and 180 Hz; on the right: (10+, 11−), 3.7 V, 90 *μ*sec, and 180 Hz. The patient was off medications at the time of the evaluation. A Spanish version [21] of the statistically validated thirteen-item Beck Depression Inventory Short Form [22, 23] (BDI-SF) was administered to the patient before changing his DBS parameters. Each question and its associated multiple choice answers were read aloud to the patient who then indicated which response best fit his current emotional state. We then changed his right-sided bipolar stimulation to the following monopolar settings: (C+, 10−), 2.5 V, 90 *μ*sec, and 180 Hz; the left side was left unchanged. Within 90 seconds, the patient became acutely dysphoric and tearful. The BDI-SF was administered a second time immediately after the onset of dysphoria. The duration of testing was approximately 15 minutes. He had some difficulty concentrating, appearing fidgety and distractible, and several questions had to be repeated. When he had finished the survey, the right-sided stimulation was changed back to the original bipolar settings, and the patient's affect returned to normal almost immediately. After ten minutes, the BDI-SF was administered for the third time. At the time of this evaluation, the patient's motor scores and optimal DBS settings had previously been determined and therefore were not repeated. The patient and administrator of the questionnaire were unblinded to the stimulation conditions.

The BDI-SF is a thirteen-item multiple choice questionnaire. Each answer is assigned a point value from 0 to 3 with 0 corresponding to least depression and 3 corresponding to most depression. The possible scores range from 0 to 39. The patient's BDI-SF scores for the first, second, and third administration were 15, 19, and 6, respectively.

## 3. Discussion

### 3.1. Evaluation of Mood State

In this study, we used a parametric psychological measurement tool before, during, and after induction of a depressive state in an attempt to gain some objective data regarding the patient's mood changes. The patient's pretest BDI-SF score of 15 is consistent with mild depression at baseline. This was not unexpected as the patient had developed some underlying depression shortly after surgery and was currently taking an antidepressant medication. Ideally, this test would have been completed soon after the patient's first DBS programming before he developed an underlying clinical depression. As it stands, we can only speculate on how much this baseline depression interacted with the induced depressive state. Subjectively from our perspective, the patient's behavior during the first event was very similar to that which was reproduced ten months later.

The increase of the score from 15 to 19 during stimulation was less dramatic than we would have anticipated given his profound change in effect. It is possible that he was simply unable to express the severity of his current dysphoric state, perhaps because of his apparent difficulty in concentrating on the questions. It is also possible that the BDI-SF may not have been the most suitable instrument for these purposes and did not accurately reflect the patient's current emotions. The dramatic decline in his posttest BDI-SF score from 19 to 6 is interesting for two reasons: first, the magnitude of the difference suggests that the depressive state was indeed severe despite the small change initially. Second, the posttest score of 6 is substantially less than the pretest score of 15 indicating less severe depression after the test than before. This is most likely a reflection of the relative change of mood state rather than an actual improvement in mood from baseline. Regarding the latter thought, the patient's affect did not seem particularly elevated after the stimulation ended; however, a transient hypomania following acute DBS-induced depression has been reported in one other case [10], thus remaining a valid possibility.

Prior studies observed the same mood phenomena and associated the patients' symptoms with a psychiatric diagnosis. Bejjani et al. reported retrospectively that their patient fulfilled seven of nine criteria for a major depressive episode [10] as defined by the Diagnostic and Statistical Manual of Mental Disorders IV (DSM-IV) [24]. Stefurak et al. noted that their patient's response was similar to major depression but was not associated with guilt or “specific negative content” [13]. Tommasi et al. had their patient evaluated by a psychiatrist during stimulation who concluded that her symptoms, though profound, did not fulfill criteria for a major depressive episode [15].

We would agree that the dysphoria experienced by these patients cannot be considered classically defined or commonly experienced depression for a couple of reasons. First, the criteria involved in DSM-IV definitions are not valid in these cases due to the short time requirement (two-week duration) as the short term dysphoria automatically precludes diagnosis. Additionally, the criteria of sleep and appetite changes do not apply in such short time periods [24]. Secondly, DBS-induced dysphoria is both qualitatively different and quantitatively more severe than the mood disturbance involved in a major depressive episode. For example, at least two patients reported feeling fearful [10, 15] which is not generally a major feature of depression. Blomstedt et al. pointed out that both theirs and Bejjani et al.'s patients described a distinctive feeling of moving into darkness [10, 14]. One patient described her acute depressive state as being “similar in some respects to my depression but a thousand times worse” [13]. The difficulty in fitting this phenomenon into an existing diagnosis suggests that it may be an entity distinct from major depression altogether.

Because the DBS-induced depressive state is so uncommon and difficult to define, objective psychological measurements are useful in providing a quantitative understanding of the acute changes in mood. With that said, the questionnaire used in this report has limitations as well. The BDI-SF is primarily used as a screening and diagnostic tool to identify patients with major depression and is not an ideal tool to indicate the acute mood state. Psychological measures that focus on current emotional state such as the Profile of Mood States (POMS) or the Visual Analogue Mood Scale (VAMS) may be more suitable in these cases.

### 3.2. Anatomical Considerations

The anatomical target for DBS in all of the described cases of transient depressive states has been the subthalamic nucleus; however, the actual locations of the responsible contacts have varied. In three of these cases, the reported locations of the electrodes were confirmed postoperatively by brain MRI. Bejjani et al. reported that the contact was located ventral to the left STN in the substantia nigra (SN) [10] while that of Stefurak et al. was found to be in the fields of Forel/zona incerta (FF/zi) region, just superolateral to the right STN [13]. Both authors attributed the depressive state to stimulation of unintended targets outside of the STN. The case reported by Tommasi et al. was interesting in this regard because it was one of the few to report this particular adverse effect with confirmed stimulation at the intended target, the STN. Additionally, due to stimulation in multiple locations, this patient was the only case reported to have this adverse effect associated with the SN and FF/zi. They also reported optimal motor improvement only with stimulation of the STN. In a fourth case, the location of the contact inducing the depressive state was inferred but not confirmed since the electrode was repositioned during surgery to have been the right SN [14]. In the remaining three cases, evidence confirming electrode positions was not reported; however, based on symptomatic motor improvements, the responsible contacts are understood to be in the subthalamic nuclei [11, 12]. Amongst these cases, Kumar et al. found that the depressive state was associated with stimulation through the contact that produced the most motor benefit [11].

Whereas many of the previous cases were associated with the most ventral contact of the DBS electrodes, the dysphoric state in our patient was induced by stimulation through the most dorsal contacts (#10 and #11). In addition, the reaction was elicited by monopolar stimulation through contacts #10 and #11 but was absent after narrowing the field with bipolar stimulation between these same contacts. This shows that the dysphoria was caused by stimulation of a region adjacent to, but not in the same place as, the dorsal end of the electrode. By contrast, optimal motor effects were produced by both monopolar and bipolar stimulation, which is consistent with accurate placement of the electrode in the STN. The more ventral contacts (#8 and #9) were not associated with mood changes but did not produce significant motor benefit either.

The STN is divided into three functional territories: the sensorimotor division which is the neurosurgical target for DBS in PD patients, the associative division which processes cognitive information, and the limbic division which processes emotional and motivational information [25]. Unintended spread of stimulation to the limbic division, which is located ventrally and medially to the sensorimotor division, provides a reasonable explanation for emotional disturbances seen in DBS patients, although the specific pathways involved have not been clearly defined. Some authors have argued for selective stimulation of the zona incerta region ventral to the STN to avoid these emotional effects [26]. Inputs to the limbic STN include the anterior cingulate and prefrontal cortices, nucleus accumbens, ventral tegmental area (VTA), and ventral pallidum; outputs include the substantia nigra and the ventral tegmental area [27].

We propose that in addition to motor benefit the monopolar stimulation of contacts situated in the lateral motor part of the STN produces an electrical field that spreads to the medial limbic STN and causes the acute depressive reaction. Once the parameters are changed to bipolar stimulation, a smaller electrical field is produced and only the lateral motor portion of the STN is being affected; therefore, the motor effects persist while the mood disturbance disappears. Conclusively, the bipolar settings that excluded the limbic STN are associated with optimal motor effects without mood disturbance.

## References

[1] Weintraub D., Burn D. J. (2011). Parkinson's disease: the quintessential neuropsychiatric disorder. *Movement Disorders*.

[2] Slaughter J. R., Slaughter K. A., Nichols D., Holmes S. E., Martens M. P. (2001). Prevalence, clinical manifestations, etiology, and treatment of depression in parkinson's disease. *Journal of Neuropsychiatry and Clinical Neurosciences*.

[3] Reijnders J. S. A. M., Ehrt U., Weber W. E. J., Aarsland D., Leentjens A. F. G. (2008). A systematic review of prevalence studies of depression in Parkinson's disease. *Movement Disorders*.

[4] Appleby B. S., Duggan P. S., Regenberg A., Rabins P. V. (2007). Psychiatric and neuropsychiatric adverse events associated with deep brain stimulation: a meta-analysis of ten years' experience. *Movement Disorders*.

[5] Temel Y., Kessels A., Tan S., Topdag A., Boon P., Visser-Vandewalle V. (2006). Behavioural changes after bilateral subthalamic stimulation in advanced Parkinson disease: a systematic review. *Parkinsonism and Related Disorders*.

[6] Kaiser I., Kryspin-Exner I., Brücke T., Volc D., Alesch F. (2008). Long-term effects of STN DBS on mood: psychosocial profiles remain stable in a 3-year follow-up. *BMC Neurology*.

[7] Funkiewiez A., Ardouin C., Caputo E. (2004). Long term effects of bilateral subthalamic nucleus stimulation on cognitive function, mood, and behaviour in Parkinson's disease. *Journal of Neurology, Neurosurgery & Psychiatry*.

[8] Funkiewiez A., Ardouin C., Cools R. (2006). Effects of Levodopa and subthalamic nucleus stimulation on cognitive and affective functioning in Parkinson's disease. *Movement Disorders*.

[9] Krack P., Batir A., van Blercom N. (2003). Five-year follow-up of bilateral stimulation of the subthalamic nucleus in advanced Parkinson’s disease. *The New England Journal of Medicine*.

[10] Bejjani B.-P., Damier P., Arnulf I. (1999). Transient acute depression induced by high-frequency deep-brain stimulation. *The New England Journal of Medicine*.

[11] Kumar R., Lozano A. M., Sime E., Halket E., Lang A. E. (1999). Comparative effects of unilateral and bilateral subthalamic nucleus deep brain stimulation. *Neurology*.

[12] Doshi P. K., Chhaya N., Bhatt M. H. (2002). Depression leading to attempted suicide after bilateral subthalamic nucleus stimulation for Parkinson's disease. *Movement Disorders*.

[13] Stefurak T., Mikulis D., Mayberg H. (2003). Deep brain stimulation for Parkinson's disease dissociates mood and motor circuits: a functional MRI case study. *Movement Disorders*.

[14] Blomstedt P., Hariz M. I., Lees A. (2008). Acute severe depression induced by intraoperative stimulation of the substantia nigra: a case report. *Parkinsonism & Related Disorders*.

[15] Tommasi G., Lanotte M., Albert U. (2008). Transient acute depressive state induced by subthalamic region stimulation. *Journal of the Neurological Sciences*.

[16] Kulisevsky J., Berthier M. L., Gironell A., Pascual-Sedano B., Molet J., Parés P. (2002). Mania following deep brain stimulation for Parkinson's disease. *Neurology*.

[17] Ulla M., Thobois S., Lemaire J.-J. (2006). Manic behaviour induced by deep-brain stimulation in Parkinson's disease: evidence of substantia nigra implication?. *Journal of Neurology, Neurosurgery and Psychiatry*.

[18] Ulla M., Thobois S., Llorca P.-M. (2011). Contact-dependent reproducible hypomania induced by deep brain stimulation in Parkinson's disease: clinical, anatomical, and functional imaging study. *Journal of Neurology, Neurosurgery and Psychiatry*.

[19] Krack P., Kumar R., Ardouin C. (2001). Mirthful laughter induced by subthalamic nucleus stimulation. *Movement Disorders*.

[20] Bejjani B. P., Houeto J. L., Hariz M. (2002). Aggressive behavior induced by intraoperative stimulation in the triangle of Sano. *Neurology*.

[21] Wiebe J. S., Penley J. A. (2005). A psychometric comparison of the Beck Depression Inventory-II in English and Spanish. *Psychological Assessment*.

[22] Furlanetto L. M., Mendlowicz M. V., Romildo Bueno J. (2005). The validity of the Beck Depression Inventory-Short Form as a screening and diagnostic instrument for moderate and severe depression in medical inpatients. *Journal of Affective Disorders*.

[23] Foelker G. A., Shewchuk R. M., Niederehe G. (1987). Confirmatory factor analysis of the short form Beck depression inventory in elderly community samples. *Journal of Clinical Psychology*.

[24] American Psychiatric Association (2000). *Quick Reference to the Diagnostic Criteria from DSM-IV-TR*.

[25] Yelnik J., Bardinet E., Dormont D. (2007). A three-dimensional, histological and deformable atlas of the human basal ganglia. I. Atlas construction based on immunohistochemical and MRI data. *NeuroImage*.

[26] Burrows A. M., Ravin P. D., Novak P. (2012). Limbic and motor function comparison of deep brain stimulation of the zona incerta and subthalamic nucleus. *Neurosurgery*.

[27] Haegelen C., Rouaud T., Darnault P., Morandi X. (2009). The subthalamic nucleus is a key-structure of limbic basal ganglia functions. *Medical Hypotheses*.

